# Tuberculosis among young contacts of patients with multidrug-resistant pulmonary tuberculosis in a reference hospital

**DOI:** 10.1016/j.jped.2025.01.008

**Published:** 2025-03-11

**Authors:** Evelyn F. Rubin, Sheila C. Lucena, Marcela Bhering, Lorrayne Gonçalves, Fabiana Falcão, Margareth Dalcolmo, Giovanni B. Migliori, Laura Saderi, Giovanni Sotgiu, Afrânio Kritski, Anna C.C. Carvalho

**Affiliations:** a Federal University of Rio de Janeiro (UFRJ), School of Medicine, Academic Tuberculosis Program, Rio de Janeiro, Brazil; b Municipal Health Secretariat of Rio de Janeiro, Municipal Hospital Raphael de Paula Souza, Rio de Janeiro, Brazil; c Oswaldo Cruz Foundation, National School of Public Health, Professor Hélio Fraga Reference Center, Rio de Janeiro, Brazil; d Oswaldo Cruz Foundation, Oswaldo Cruz Institute, Laboratory of Innovations in Therapies, Education and Bioproducts (LITEB), Rio de Janeiro, Brazil; e Maugeri Clinical Institutes IRCCS, Maugeri, Clinical Epidemiology Service for Respiratory Diseases, Italy; f University of Sassari, Department of Medical, Surgical and Experimental Sciences, Clinical Epidemiology and Medical Statistics Unit, Sassari, Italy

**Keywords:** Tuberculosis, Multidrug-resistant tuberculosis, Contact tracing, Children, Adolescent

## Abstract

**Objectives:**

Young contacts of pulmonary tuberculosis (TB) patients face a higher risk of TB. Still, few studies have evaluated this risk among contacts of patients with pulmonary multidrug-resistant tuberculosis (MDR-TB). This study aimed to describe the incidence rate and the prevalence of TB infection (TBI) and TB disease (TBD) in young contacts of patients with MDR-TB.

**Methods:**

The authors retrospectively evaluated contacts of patients with pulmonary TB aged 0 to 19 for TBI and TBD in Rio de Janeiro between 2006 and 2016. Based on the drug susceptibility pattern and/or therapeutic regimen of the index case, contacts were classified into MDR-TB and non-MDR-TB contacts. A tuberculin skin test ≥ 5 mm was considered positive. Preventive therapy with isoniazid was offered to eligible contacts. Bivariate and multivariate logistic regressions estimated factors associated with TBI.

**Results:**

439 contacts were screened; 129 were MDR-TB and 310 were non-MDR-TB contacts. TBI prevalence was 68.2 % in MDR-TB vs. 61.9 % in non-MDR-TB contacts (*p* = 0.23). Tuberculin conversion was higher among MDR-TB contacts (45.5 % vs. 17.1 %; *p* = 0.04). TBD incidence rate was 47.7 in non-MDR-TB and 179.6 per 100,000 person-months in MDR-TB contacts (*p* = 0.65), for a total TBD prevalence of 2.5 %. The overall TPT completion rate was 67.2 %; 71.5 % in non-MDR-TB and 59 % in MDR-TB contacts (*p* = 0.04).

**Conclusion:**

The authors identified a high prevalence of TBI among contacts of pulmonary MDR-TB and non-MDR-TB patients, with a higher tuberculin conversion rate in MDR-TB contacts, highlighting the urgency of effective TPT regimens for young contacts of patients with pulmonary MDR-TB.

## Introduction

The presence of *Mycobacterium tuberculosis* (MTB) strains resistant to the most effective anti-tuberculosis (TB) drugs poses a significant threat to the elimination of TB.^1^^,^^2^ Globally, it is estimated that 400,000 people were diagnosed with rifampicin-resistant TB (RR-TB) or with multidrug-resistant TB (MDR-TB, i.e., resistant to at least rifampicin and isoniazid).^2^ In Brazil, between 2015 and 2023, 17,200 new cases of drug-resistant TB (DR-TB) were reported; of these, 1,060 were diagnosed in 2023, representing an increase of 6 % compared to those diagnosed in 2018.^3^

Nearly a quarter of the world's population is estimated to be infected with MTB.^4^ Children under 5 years old are at a significantly higher risk of developing TB disease (TBD) after TB infection (TBI), with a cumulative incidence approaching 20 % within 2 years of exposure, and are more likely to present with severe forms of TB disease.^5^

The detection of TBI and its preventive treatment (TPT) with isoniazid and/or rifamycins among close contacts of patients with drug-susceptible pulmonary TB represents a public health priority.^2^^,^^6^ However, when the present study was carried out, no TPT regimen had been widely validated for contacts of patients with MDR-TB. In 2024, the World Health Organization (WHO) included 6 months of daily levofloxacin as a TPT option for people exposed to MDR/RR-TB in the WHO guidelines for TPT^7^ (replacing the previously proposed conditional recommendation)^8^ based on the results of two clinical trials (VQUIN MDR and The TB-CHAMP) that were recently published.^9^^,^^10^ However, the final results of these studies, although they have demonstrated a lower percentage of TBD in the levofloxacin-treated group than in the placebo group, the difference found was not statistically significant.

In Brazil, few studies have assessed the prevalence of TBI and TBD in contacts of patients with MDR-TB, with a small proportion of children and adolescents evaluated.^11^^,^^12^ The present study aimed to assess the prevalence of TBI and TBD in children and adolescent contacts of patients with drug-susceptible and drug-resistant pulmonary TB, as well as the incidence of TBD among contacts exposed to isoniazid preventive therapy.

## Material and methods

### Data and sample

This retrospective cohort study included contacts aged 0 to 19 years old who were screened for TBD and TBI at the pediatric pneumology outpatient clinic of the Municipal Hospital Raphael de Paula Souza, a reference center for screening young contacts of patients with MDR/XDR-TB in Rio de Janeiro. The state of Rio de Janeiro stands out as having the third-highest TB incidence and the second-highest TB mortality rate in Brazil, recorded, respectively, at 70.7 in 2023 and 4.7 per 100,000 inhabitants in 2022. Additionally, Rio de Janeiro accounts for 15 % of all notified DR-TB patients in the country.^3^

Eligible contacts underwent their first assessment from January 2006 to December 2016, and the last follow-up visit occurred before January 2019. Children and adolescents who lived in the same household or had close contact with a patient with pulmonary TB (henceforth named “index case”) and underwent at least one tuberculin skin test (TST) were eligible for the study. Information regarding index cases (clinical and radiological data, microbiological testing, including sputum smear microscopy, culture, and rapid molecular testing, and drug susceptibility testing-DST results) was retrieved from contact referral forms and the Tuberculosis Special Treatment Notification System (SITE-TB).^6^

Contacts whose index cases did not have clinical/laboratory information available to define if they had MDR or non-MDR pulmonary TB, contacts without TST results, and those whose contact with the index case occurred >2 years before the first visit were excluded. All children and adolescents recruited for the study were evaluated and followed by the same medical professional (SCL), who was responsible for the pediatric TB outpatient clinic at Raphael de Paula e Souza Hospital. EFR and SCL jointly reviewed all the medical records.

### Operational definitions of key terms

Index case: patient with pulmonary TB from whom the contact assessment was carried out. Contacts were classified according to the pattern of drug resistance to anti-TB drugs or treatment regimens prescribed for the index case as MDR-TB and non-MDR-TB contacts. The diagnosis of TB in the index case was based on a positive rapid molecular test (Xpert MTB/RIF; Cepheid, Sunnyvale, CA, USA) and/or spontaneous, induced, or bronchoalveolar lavage sputum culture of MTB.^6^

MDR-TB contact: contacts of patients with pulmonary TB caused by MTB strains resistant to at least rifampicin and isoniazid, including patients with extensively drug-resistant TB (XDR-TB; i.e., MDR-TB plus resistance to a second-line injectable drug and a fluoroquinolone) and pre-extensively drug-resistant TB (pre-XDR, i.e. MDR-TB with additional resistance to either a second-line injectable drugs and/or any fluoroquinolone), according to the definitions of XDR-TB at the time of the study.^6^

Non-MDR-TB contact: contacts of patients with pulmonary TB whose DST showed susceptibility to all first-line drugs, resistance profile different from MDR-TB, or, in the absence of DST, when the index case had a clinical and radiological response to the standard first-line TB treatment.

Positive TST: an induration ≥ 5 mm 48–72 hours after TST using 2 IU of PPD-RT23 (Statens Serum Institut, Copenhagen/Denmark), applied by the Mantoux method.^13^

Tuberculin conversion: defined as an increase of at least 10 mm in skin induration on a second TST performed 8 weeks after the first negative TST.^13^

Tuberculosis preventive therapy (TPT): 6-months isoniazid treatment was offered to all asymptomatic contacts with positive TST, normal physical examination, and negative findings on the chest radiograph. Contacts considered at high risk of TB progression by the attending physician received isoniazid regardless of TST result_._^13^

Tuberculosis infection (TBI): contacts without clinical symptoms or laboratory results compatible with TBD, who had a TST result equal to or greater than 5 mm and a normal chest radiograph.^13^

Tuberculosis disease (TBD): the presence of clinical and radiographic findings suggestive of active TB, as well as a positive TST result, as described in the clinical scoring system of the Brazilian Ministry of Health (MoH).^6^ For children and adolescents without microbiological confirmation, the score obtained in the MoH scoring system was used to define TB cases, as follows: 40 points (very likely diagnosis); 30-35 points (possible diagnosis); and <25 points (diagnosis is unlikely). For cases in which it was possible to collect a biological sample, the presence of acid-fast bacilli on direct examination, positive Xpert MTB/RIF result, and/or positive MTB culture were considered confirmed TBD cases.

Co-prevalent tuberculosis disease: contacts diagnosed with TBD up to 8 weeks from the index case's first medical visit.

Incident tuberculosis disease: contacts diagnosed with TBD after 8 wk from the first medical visit.

Follow-up group: contacts of patients with pulmonary MDR-TB without TBD at the first evaluation who attended at least two medical visits with an interval of more than one week between them.

### Statistical analysis

Qualitative variables were summarized as absolute and relative frequencies and compared using the chi-square or Fisher's exact test. Quantitative variables were described as medians (interquartile ranges) due to their non-parametric distribution, evaluated by the Shapiro-Wilk test, and compared using the Mann-Whitney test.

The overall prevalence of TBI was calculated using TST-positive results at baseline and after 8 weeks as the numerator.

TBD incidence rates (per 100,000 person-months) were calculated in the MDR-TB and non-MDR-TB groups and overall, with incident TB patients as the numerator and the total number of person-month contacts as the denominator, as well as for groups according to completion or non-completion-of-preventive therapy. Incidence rate ratios (IRRs) with 95 % confidence intervals (CIs) were calculated in both cases.

Bivariate logistic regressions were performed to assess the association between TBI and independent variables. Variables with significance levels ≤0.20 in univariate analysis were included in multivariate logistic regression models. A *p*-value <0.05 was adopted to define a statistically significant difference. STATA version 16 software (StatsCorp, Texas, USA) was used for all calculations.

### Ethical approval statement

The study protocol was approved by the Research Ethics Committee of Instituto Oswaldo Cruz (CAAE 3007 1420 0 0000 5248), Fiocruz, and successively by the Municipal Health Secretary of Rio de Janeiro, which granted permission for the use of the identified data for the study and waived the need for written informed consent from participants as the study was based on secondary data and involved no more than minimal risk. All patients had an identification number, and to protect patients' confidentiality, only two investigators (EFR and SCL) had access to both identified and de-identified codes; EFR prepared the anonymous database that was used in the study.

## Results

### Sociodemographic and clinical characteristics of contacts

Out of 529 contacts evaluated during the study period, 14 (2.6 %) were excluded due to a lack of data on the index case. Among the remaining 515 contacts, TST was not performed in 69 (13.4 %), and 7 (1.6 %) did not return for TST reading, resulting in their exclusion from the study. A total of 439 contacts were included, corresponding to 215 index cases (138 patients with non-MDR-TB and 77 patients with MDR-TB). The HIV serology result was known in 130/215 (60.4 %) of index cases, of whom 25.4 % (33/130) resulting positive.

Among contacts, 310 (70.6 %) were contacts of patients with non-MDR-TB, and 129 (29.4 %) were contacts of patients with MDR-TB. In both groups, most index cases were parents (53.5 %). Index cases with MDR-TB had a higher frequency of sputum smear positivity, cavitations, and bilateral disease on chest radiography. DST resistance results for at least one drug were available for 34.3 % (151/439) of contacts. Among MDR-TB contacts, the most common resistance pattern was MDR-TB (77.5 %), and for non- MDR-TB contacts, the most common was polyresistance (72.2 %) (Table 1).

**Table 1:** Sociodemographic and clinical characteristics of 439 children and adolescent contacts by drug resistance pattern of index cases (non-MDR-TB and MDR-TB).

	Contacts -total	Non-MDR-TB	MDR -TB	*p*-value^a^
	*n* = 439 (%)	*n* = 310 (%)	*n* = 129 (%)	
*Contacts characteristics*				
Sex				0.06
Female	225 (51.2)	150 (48.4)	75 (58.1)	
Male	214 (48.8)	160 (51.6)	54 (41.9)	
Age; years (Median [IQR]^b^)	7 [3–10]	7 [3–10]	6 [3–10]	0.49
Age range				0.95
0–4	158 (36.0)	110 (35.5)	48 (37.2)	
5–9	148 (33.7)	107 (34.5)	41 (31.8)	
14-Oct	115 (26.2)	80 (25.8)	35 (27.1)	
15–19	18 (4.1)	13 (4.2)	5 (3.9)	
BCG scar (*n* = 436)				
Absent	9/436 (2.1)	7/308 (2.3)	2/128 (1.6)	0.63
Present	427/436 (97.9)	301/308 (97.7)	126/128 (98.4)	
Previous TB treatment	1/439 (0.2)	1 (0.3)	0 (0.0)	1
Previous TPT	7/439 (1.6)	1 (0.3)	6 (4.7)	0.003
Symptom at the 1st consultation				
Cough	50/420 (11.9)	41/297 (13.8)	9/123 (7.3)	0.06
Fever	14/420 (3.3)	10/297 (3.4)	4/123 (3.3)	0.95
Weight loss	5/420 (1.2)	5/297 (1.7)	0/123 (0.0)	0.33
Lymphadenopathy	5/420 (1.2)	3/297 (1.0)	2/123 (1.6)	0.63
Comorbidities^a^	40/434 (9.2)	30/308 (9.7)	10/126 (7.9)	0.72
HIV status				
Negative	44/45 (97.8)	34/35 (97.1)	10/10 (100.0)	1
Positive	1/45 (2.2)	1/35 (2.9)	0/10 (0.0)	
*Index case characteristics*				
Relationship with the contact				0.02
Parent	235/439 (53.5)	158/310 (51.0)	77/129 (59.7)	
Siblings	28/439 (6.4)	25/310 (8.1)	3/129 (2.3)	
Grandparents	66/439 (15.0)	42/310 (13.6)	24/129 (18.6)	
Others	110/439 (25.1)	85/310 (27.4)	25/129 (19.4)	
Same household	358/381 (94.6)	234/256 (91.4)	124/125 (99.2)	0.002
Slept with child	54/336 (16.1)	34/238 (14.3)	20/98 (20.4)	0.17
Smear positivity	370/398 (93.0)	243/269 (90.3)	127/129 (98.5)	0.003
Pulmonary cavitation	140/163 (85.9)	27/42 (64.3)	113/121 (93.4)	<0.0001
Pulmonary form				0.01
Unilateral	39/161 (24.2)	16/40 (40.0)	23/121 (19.0)	
Bilateral	122/161 (60.0)	24/40 (60.0)	98/121 (81.0)	
Type of drug resistance				
Primary resistance	21/144 (14.6)	4/22 (18.2)	17/122 (13.9)	
Acquired resistance	123/144 (85.4)	18/22 (81.8)	105/122 (86.1)	0.53
Pattern of drug-resistance (DST)^c^				<0.0001
Mono^d^	5/151 (3.3)	5/22 (22.7)	0/129 (0.0)	
Poly^e^	16/151 (10.6)	16/22 (72.7)	0/129 (0.0)	
MDR	117/151 (77.5)	0/22 (0.0)	117/129 (90.7)	
XDR	12/151 (8.0)	0/22 (0.0)	12/129 (9.3)	
RR	1/151 (0.7)	1/22 (4.6)	0/129 (0.0)	
HIV positivity	30/152 (19.7)	17/47 (36.2)	13/105 (12.4)	0.002

TB, tuberculosis; MDR-TB, multidrug-resistant tuberculosis; TPT, tuberculosis preventive treatment.

a Comparison between non-MDR and MDR contacts.

b Median [IQR 25–75];*26 different comorbidities were recorded; asthma was the most common (35 %; 14/40).

c Information on the number of index cases that underwent DST or the results of DST for drug-sensitive patients was not available. In these cases, the contact classification in MDR or non-MDR-TB was based on information on the treatment regimen adopted by the index case and the respective therapeutic response collected.

d Mono = resistance to one drug only (5 resistant to Isoniazid).

e Poly = resistance to two or more drugs except to both rifampicin and isoniazid (1 Rifampicin+Ethambutol; 7 Isoniazid+Streptomycin; 2 Rifampicin+Streptomycin; 2 Isoniazid+Pyrazinamide+Ethambutol; 3 Isoniazid +Ethambutol; 1 Isoniazid +Ethambutol+Amikacin+Ofloxacin).

### Tuberculosis infection

**Figure 1:**
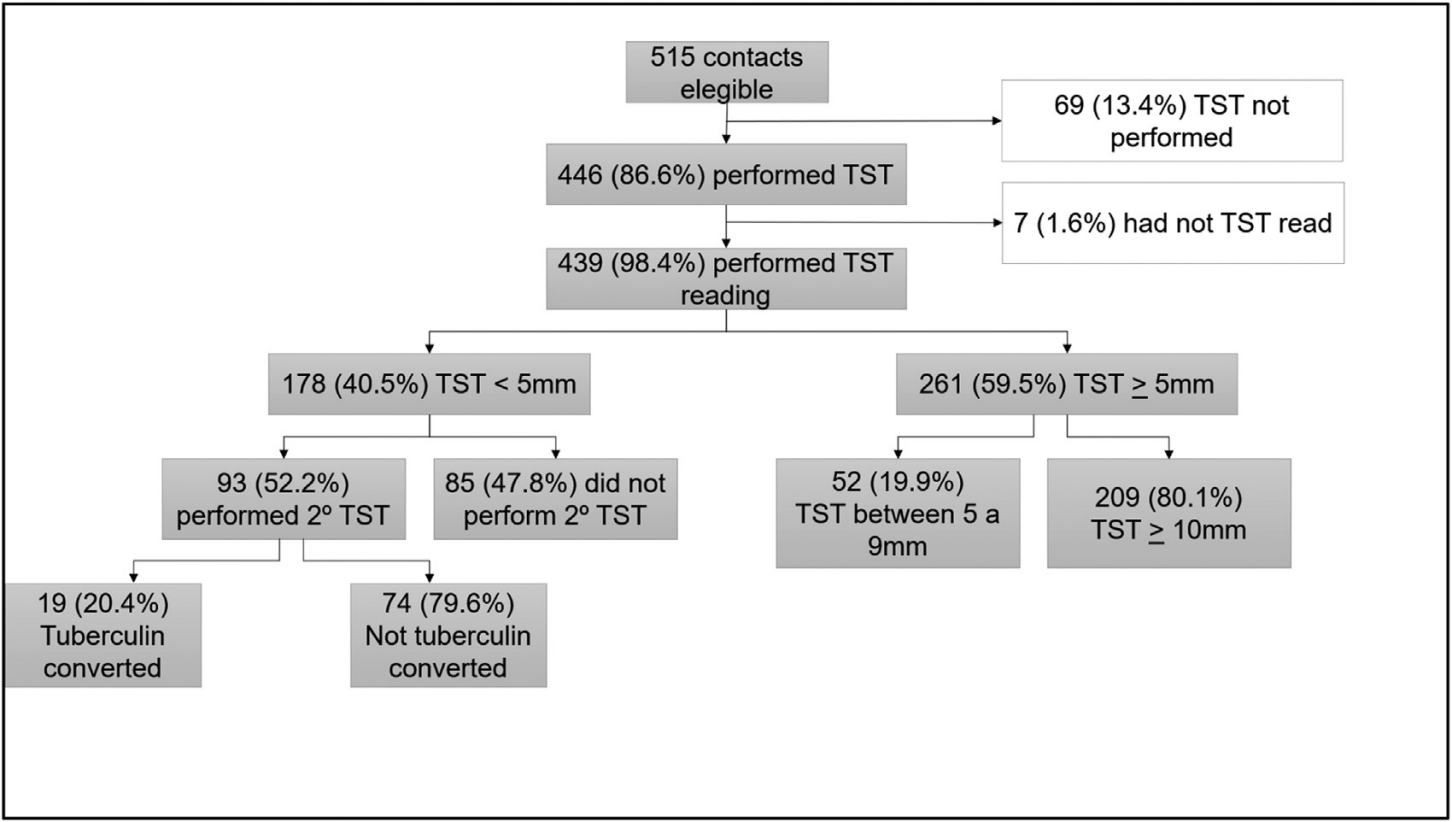
Flowchart of tuberculin skin test (TST) results among children and adolescents contacts of patients with non-MDR-TB and MDR-TB.

Figure 1 presents the TBI evaluation flow among contacts. TST positivity at the first assessment was 59.5 % (261/439). Among the 178 contacts with an initial negative TST, 93 (52.2 %) underwent a second TST after 8 weeks. Early tuberculin conversion was observed in 19 out of 93 individuals (20.4 %). The final prevalence of TBI was 63.8 % (280/439).

Tuberculin conversion was significantly higher in MDR-TB contacts (45.5 % vs. 17.1 %; *p* = 0.04). However, the percentage of initially negative TST contacts who returned for a second TST was lower among MDR-TB than non-MDR-TB contacts (24 % vs. 62 %, respectively). Considering the combined TST results, TBI prevalence was slightly higher in contacts of MDR-TB patients, but the difference between groups was not statistically significant (68.2 % vs. 61.9 %, *p* = 0.23) (Table 2).

**Table 2:** Tuberculin skin test (TST) response of children and adolescents who are a close contact of non-MDR-TB and MDR-TB patients.

Variables	Non MDR-TB n (%)	MDR-TB n (%)	OR (95 % CI)	*p*-value
Initial TST response				
< 5 mm	132 (42.6)	46 (35.7)	1	0.20
≥ 5 mm	178 (57.4)	83 (64.3)	1.34 (0.87–2.05)	
Second TST - tuberculin conversion				
Yes	14 (17.1)	5 (45.5)	4.05 (1.08–15.13)	0.04
No	68 (82.9)	6 (54.5)		
TBI prevalence^a^				
TST - positive	192 (61.9)	88 (68.2)	1.32 (0.85–2.04)	0.23
TST - negative	118 (38.1)	41 (38.8)		

MDR-TB, multidrug-resistant tuberculosis; TST, tuberculin skin test.

a Combined TST results.

### Tuberculosis disease

At the initial assessment, 1.6 % (7/439) of contacts were diagnosed with TBD and were classified as co-prevalent cases. 411 contacts (TST-positive and TST-negative) were followed for a median of 30 weeks (IRQ 20 to 45), and 4 additional contacts (0.97 %) developed TB, representing incident cases. Combined, these accounted for 2.5 % of the cohort diagnosed with TBD, with 2.26 % (7/310) in the non-MDR-TB group and 3.1 % (4/129) in the MDR-TB group. The incidence rate of TBD among contacts MDR-TB patients was 179.6 per 100,000 person-months vs. 47.7 per 100,000 person-months for contacts of non-MDR-TB patients, resulting in an incidence rate ratio (IRR) of 3.76 (95 % CI: 0.30–197.2); however this difference was not statistically significative (*p* = 0.27).

Microbiological confirmation of TBD was available for only 2 of 11 contacts (18.2 %). Ten of 11 contacts (91 %) had pulmonary TB. The median age was 7.0 years, and three children were younger than 5 years old. Among 8 children tested, all were HIV seronegative. DST was available in only one contact of a patient with MDR-TB, who presented a different DST pattern and had secondary resistance to anti-TB drugs.

Among 11 contacts with TBD, 5 had previously started TPT with isoniazid; 2 were already on TPT at the baseline visit and 3 had started TPT after the first visit but were lost to the follow-up during TPT, returning to medical care after the onset of TB symptoms. DST results were available only for one contact of a patient with non-MDR-TB who developed TBD by a fully sensitive MTB strain during follow-up. Treatment was successful for seven children, one was lost to follow-up, and three were transferred out (Table S1 and S2 in the Supplementary Material).

### TB preventive therapy

**Figure 2:**
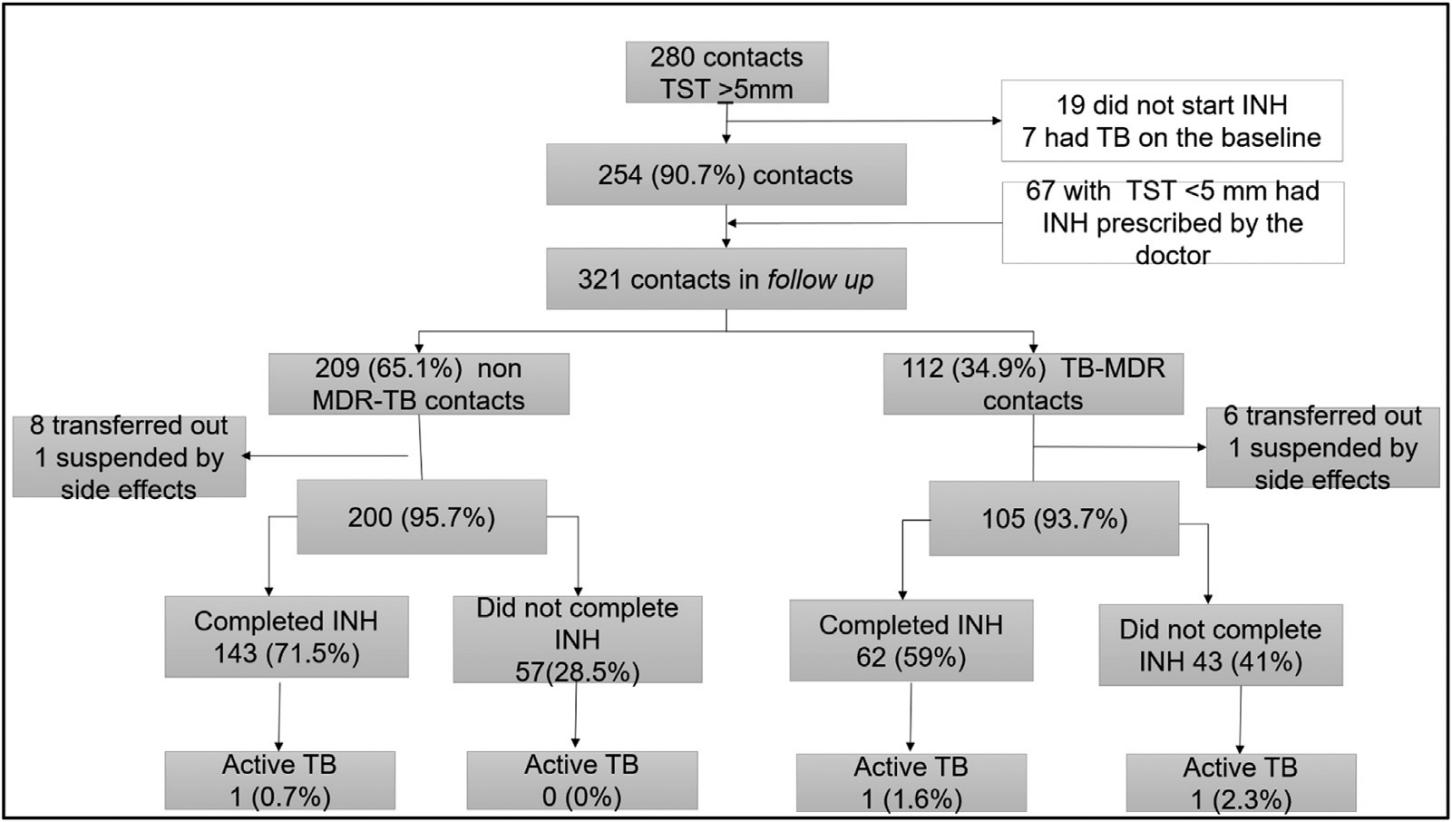
Flowchart of isoniazid preventive therapy among children and adolescents contacts of non-MDR-TB and MDR-TB patients. INH, isoniazid; TST, tuberculosis skin test. One contact of patient with MDR-TB developed TBD and did not start TPT.

Among the 321 contacts who initiated preventive therapy with isoniazid, 254 (79.1 %) had TST-positive results, while 67 (20.9 %) were considered at high risk for TBD, including 35 children under 5, who initiated TPT even presenting TST-negative results. Of these, 209 (65.1 %) were contacts of patients with non-MDR-TB, and 112 (34.9 %) of MDR-TB (Figure 2).

No significant differences were found for the occurrence of adverse events or time of exposure to isoniazid between the groups of contacts. The overall adherence was 67.2 % (205/305), but it was significantly higher among non-MDR-TB contacts: 71.5 % (143/200) vs. 59.0 % (62/105) (*p* = 0.04).

Three contacts developed TBD after preventive therapy initiation with isoniazid; 2 of these contacts were in the MDR-TB group, and both index cases had secondary resistance. The TB incidence rate was not significantly higher among MDR-TB contacts who did not complete preventive therapy, however, the sample size was too small (Supplementary material S3). The contact of a patient with RR-TB received TPT with isoniazid and did not develop TBD during the follow-up period.

Regarding the association between TBI and the independent variables in the final multivariate model, the risk of TBI increased with the age of contact (OR 1.13; 95 % CI 1.03–1.25) and decreased when the index case was a grandparent (OR 0.33; 95 % CI 0.12–0.93) or HIV-positive (OR 0.28; 95 % CI 0.12–0.69). The infectivity of the index case (assessed by sputum smear positivity and the presence of cavitation on the chest radiograph), as well as the presence of resistance to anti-TB drugs, were not associated with TBI among contacts in the final model (Supplementary material S4).

## Discussion

In Brazil, the risk of TB infection and disease among young contacts of patients with pulmonary MDR/XDR-TB is not well known.^6^^,^^11^^,^^12^ In the present study, the authors found a high prevalence of TBI among contacts (59.5 %) at the initial assessment, which rose to 63.8 % after the second TST. These findings are similar to those reported in low and middle-income countries, which showed a spectrum of TB infection prevalence ranging from 57 % to 72 % amidst both pediatric and adult contacts of patients with MDR-TB,^14^^,^^15^ whereas contacts of individuals with drug-susceptible TB exhibited rates ranging from 44 % to 83 %.^16^^,^^17^

A significant proportion of tuberculin conversion (TC) was observed (20.4 %), as described in a previous study carried out in the same city^16^, emphasizing the importance of a second TST for initially TST-negative contacts. TC was higher among MDR-TB contacts (45.5 % vs. 17.7 %), possibly due to prolonged exposure to a symptomatic index case,^18^ but this finding may have been biased by the different proportion of contacts who returned for the second TST in each group. However, recently it has been described that the risk of acquiring TBI and developing TBD among contacts of patients with RR/MDR-TB may persist for up to a year, despite index case treatment and evaluation of contacts for TBI and TBD at baseline.^19^

While early studies suggested attenuated pathogenicity of drug-resistant MTB strains,^20^^,^^21^ later research by Snider et al. found no differences in transmissibility between drug-susceptible and resistant strains.^22^ A limited number of prospective studies indicate that transmissibility depends on specific genomic mutations.^23^

Most research on pediatric and adolescent contacts of patients with drug-resistant TB has been conducted in high TB-HIV co-infection settings, such as South Africa.^24^

Among contacts, HIV infection increases the risk of TB infection and disease.^5^^,^^15^ In this study, only 10 % of contacts were tested for HIV, with one (2.2 %) tested positive.

The authors did not find a statistically significant difference in the risk of TBD between contact groups. Several studies on the incidence of secondary TBD among household contacts with MDR-TB have had low statistical power or did not include a control group.^11^^,^^12^^,^^25^ These findings are similar to previous studies that reported TBD rates of 1.2 % to 7.8 % among MDR contacts.^11^^,^^22^^,^^26^

In the present study, TPT initiation with INH was lower among MDR-TB contacts (34.9 % vs 65.1 %), as well as the adherence to TPT (59.0 % vs. 71.5 %). Additionally, three out of four individuals who developed incident TBD were MDR-TB contacts. Contacts of MDR-TB patients who did not complete TPT had a higher risk of TB, though the small sample size limits the reliability of this finding. Similarly, Kritski et al. reported TBD in 4 % of contacts receiving isoniazid-based TPT compared to 9 % of untreated contacts.^11^ However, their study focused on adult contacts, where prior exposure to isoniazid-sensitive TB may have influenced outcomes. A Peruvian study found that TPT with isoniazid-protected contacts under 20 years of age against pulmonary MDR-TB (HR 0.19; 95 % CI 0.05–0.66), but not against mono–isoniazid-resistant TB (HR 0.80; 95 % CI 0.23–2.80).^25^

Despite the lower adherence to TPT among MDR-TB contacts, the authors may consider that TPT completion rates in this study were moderate to high. A meta-analysis reported completion rates near 90 % in research centers,^27^ however, in routine practice, completion rates tend to be lower, with fewer than half of individuals who start TPT completing treatment in some settings.^28^^,^^29^

Considering the retrospective design of the present study, based on medical records and electronic notification records, the authors faced limitations in the completeness of the information, particularly concerning data from index cases (such as HIV serological status, chest radiograph findings or DST results), since these patients were followed up in other medical services. However, the same physician evaluated and monitored all contacts and, together with another doctor, reviewed all the medical records and filled out the forms, ensuring the standardized application of the contact evaluation protocol and, therefore, increasing data quality.

In conclusion, the authors found a high prevalence of TBI among children and adolescents who were contacts of pulmonary MDR and non-MDR-TB patients, with a higher tuberculin conversion rate among MDR-TB contacts. Nevertheless, TBI and TBD prevalence did not significantly differ between MDR-TB and non-MDR-TB contact groups.

The results reinforce the need for timely assessment of contacts of patients with MDR-TB and the provision of TPT with effective treatment regimens, which still need to be defined and effectively implemented.

## Conflicts of interest

The authors declare no conflicts of interest.

## Appendix Supplementary materials

Image, application 1
