# Supplementary material for: Tuberculosis among young contacts of patients with multidrug-resistant pulmonary tuberculosis in a reference hospital
Source: J Pediatr (Rio J). 2025 Mar 11;101(3):458–65. doi: 10.1016/j.jped.2025.01.008 (PMC12039512; doi:10.1016/j.jped.2025.01.008)
Supplement: Supplementary file 1 [file mmc1.docx]

**JPED-D-24-00343_Supplementary material**

**Table S1** Clinical characteristics of non-MDR-TB and MDR-TB contacts with TB disease at the baseline.

| **Patients** | **Clinical form** | **Sex** | **Age** | **HIV** | **TST**  **result**  **(mm)** | **TPT** | **TPT outcome** | **TB diagnosis**  **criteria** | **MDR-TB IC** | **Resistance profile of MDR-TB**  **IC** | **Outcome** |
| --- | --- | --- | --- | --- | --- | --- | --- | --- | --- | --- | --- |
| 1 | Pulmonary | M | 2 | Negative | 16 | No | --- | MH Score | No | ----- | Transferred out |
| 2 | Pulmonary | F | 6 | Negative | 0 | No | --- | Culture (+), DTS not done | No | ----- | Transferred out |
| 3 | Pulmonary | M | 6 | Negative | 13 | No | --- | MH Score | No | ------ | Treatment  completed |
| 4 | Pulmonary | F | 10 | Negative | 12 | No | --- | MH Score | No |  | Treatment  completed |
| 5 | Cervical lymph node | F | 7 | Negative | 22 | Yes | Lost to follow up | MH Score | Yes | R-H  (secondary resistance) | Treatment  completed |
| 6 | Pulmonary | M | 12 | Not done | 18  TC* | Yes | Switch from INH to RHZ | MH Score | No | ------- | Treatment  completed |
| 7 | Pulmonary | M | 8 | Negative | 12 | No | ---- | MH Score | No | ------- | Treatment  completed |

TB, tuberculosis; MDR-TB, multidrug-resistant tuberculosis; TST, Tuberculin Skin Test; TPT, TB preventive therapy; MH, Ministry of Health; IC, index cases; DST, drug sensitivity test; TC, tuberculin conversion; INH, isoniazid; RHP, rifampicin, isoniazid, pirazinamide.

| **Patients** | **Clinical form** | **Sex** | **Age** | **HIV** | **TST**  **result**  **(mm)** | **TPT** | **TPT outcome** | **TB Diagnosis** | **MDR-TB IC** | **Resistance profile of MDR-TB**  **IC** | **Outcome** |
| --- | --- | --- | --- | --- | --- | --- | --- | --- | --- | --- | --- |
| Case 1 | Pulmonary | M | 15 | Not done | 12 | No | --- | MH Score | Yes | R-H-S-Z  (secondary resistance) | Transferred out |
| Case 2 | Pulmonary | F | 8 | Negative | 5 | Yes | Lost to follow up | Culture/DST (Sensitive to R,H,E,Km, Cm,Am and Ofx) | Yes | R-H  (secondary resistance) | Lost to follow up |
| Case 3 | Pulmonary | M | 1 | Not done | 10 | Yes | Lost to follow up | MH Score | No | ------ | Treatment  completed |
| Case 4 | Pulmonary | F | 1 | Negative | 0 | Yes | Lost to follow up | MH Score | Yes | R-H  (secondary resistance) | Treatment  completed |

**Table S2** Clinical characteristics of non MDR-TB and MDR-TB contacts with TB disease during the follow-up.

TB, tuberculosis; MDR-TB, multidrug-resistant tuberculosis; TST, Tuberculin Skin Test; TPT, TB preventive therapy; MH, Ministry of Health; DST, drug sensitivity test; R, rifampicin; H, isoniazid; E, ethambutol; Km, kanamycin; Cm, capreomycin; Am, amikacin; Ofx, ofloxacin.

**Table S3** Incidence rate ratio of TB disease according to INH preventive therapy among MDR-TB contacts.

|  | **INH preventive therapy not completed** | **INH preventive therapy** | **Total** |
| --- | --- | --- | --- |
| Active TB, n | 1 | 2 | 3 |
| Person-months | 162 | 1,518 | 1,680 |
| Incidence rate, per 100,000 person-months | 617.3 (30.9-3,044.0) | 131.8 (22.1-435.3) | 178.6 |
| **IRR (95% CI):** 0.21 (0.01-12.59), p-value: 0.29 | | | |

INH, isoniazid; IRR, incidence rate ratio; CI, confidence interval.

**Table S4** Logistic regression analysis of variables associated with tuberculosis infection among contacts of patients with MDR and non-MDR pulmonary tuberculosis.

| **Characteristics** | **Univariable analysis** | | **Multivariable analysis** | |
| --- | --- | --- | --- | --- |
|  | **OR (95% CI)** | **p-value** | **OR (95% CI)** | **p-value** |
| MDR-TB contact | 1.32 (0.85-2.04) | 0.21 |  |  |
| Male | 1.40 (0.95-2.07) | 0.09 | 1.05 (0.49-2.23) | 0.90 |
| Age, years | 1.11 (1.06-1.17) | <0.0001 | 1.13 (1.03-1.25) | 0.009 |
| IC relationship to the contact  Parent  Siblings  Grandparents  Others | Ref  0.63 (0.28-1.39)  0.53 (0.30-0.92)  0.79 (0.49-1.27) | Ref  0.25  0.03  0.33 | Ref  -  0.33 (0.12-0.93)  1.30 (0.52-3.28) | Ref  -  0.04  0.57 |
| BCG vaccination | 6.33 (1.30-30.86) | 0.02 | 2.21 (0.22-22.16) | 0.50 |
| Comorbidities | 0.66 (0.34-1.27) | 0.21 |  |  |
| Contact chest radiograph findings at baseline | 1.07 (0.64-1.77) | 0.80 |  |  |
| IC home contact | 2.10 (0.90-4.91) | 0.09 | - | - |
| IC slept with child | 1.15 (0.63-2.11) | 0.64 | - | - |
| IC smear positivity | 2.58 (1.19-5.62) | 0.02 | 2.54 (0.19-34.4) | 0.48 |
| IC pulmonary cavities | 1.87 (0.77-4.57) | 0.17 | - | - |
| IC drug resistance | 1.35 (0.89-2.04) | 0.16 | - | - |
| IC rifampicin resistance | 1.29 (0.24-6.93) | 0.76 |  |  |
| IC pre-XDR | 0.38 (0.10-1.50) | 0.17 |  |  |
| IC XDR | 0.39 (0.12-1.23) | 0.11 | - | - |
| HIV positivity | 0.37 (0.16-0.82) | 0.02 | 0.28 (0.12-0.69) | 0.006 |

IC, index case; OR, odds ratio; CI, confidence interval.
